# Vacuum-Assisted Gas Stripping Extractive Fermentation for Process Intensification of Sorghum-Based Bioethanol Production: An Experimental and Modeling Study

**DOI:** 10.1021/acsomega.6c01889

**Published:** 2026-06-29

**Authors:** Letícia P. Almeida, Mateus N. Esperança, Antonio J. G. Cruz, Mark R. Wilkins, Alberto C. Badino

**Affiliations:** † Graduate Program of Chemical Engineering, Federal University of São Carlos, São Carlos, SP 13565-905, Brazil; ‡ 119519 Federal Institute of Education, Science, and Technology of São Paulo, Campus Capivari, Capivari, SP 13365-010, Brazil; § Carl and Melinda Helwig Department of Biological and Agricultural Engineering, Kansas State University, Manhattan, Kansas 66506, United States

## Abstract

Extractive fermentation can mitigate the inhibitory effects of ethanol on yeast cells by continuously removing ethanol as it is produced, thereby enhancing ethanol productivity during the sorghum hydrolysate fermentation. This approach represents a promising strategy for process intensification in starch-based bioethanol production, particularly in systems limited by product inhibition. The present study describes the modeling and experimental validation of ethanol production in separate hydrolysis and fermentation (SHF) and simultaneous saccharification and fermentation (SSF) processes, utilizing vacuum-assisted gas stripping for in situ ethanol removal. Initially, kinetic parameters for saccharification and fermentation were determined separately. Subsequently, SSF experiments were conducted to evaluate the predictive capability of the proposed model. Finally, extractive SHF and SSF experiments were carried out in a 2–liter bubble column bioreactor under vacuum-assisted gas stripping conditions. The proposed model for SHF and SSF processes, based on mass balances for liquefied starch (St), glucose (G), and ethanol (E), and incorporating saccharification and fermentation kinetics with product inhibition, showed strong agreement with the experimental data for both conventional and extractive fermentations, achieving R^2^ values above 0.99. Furthermore, vacuum-assisted gas stripping proved to be an effective process intensification strategy, increasing ethanol productivity by up to 60%. These results highlight its potential for improving ethanol production from starch-based substrates.

## Graphical abstract

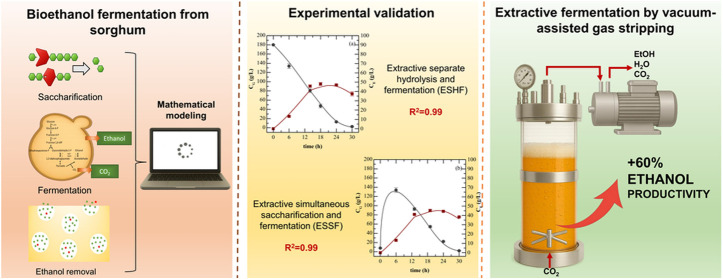

## 1 Introduction

Concerns about climate change, driven by greenhouse gas emissions and the depletion of fossil fuel-based energy sources such as oil, coal, and natural gas, have led to an increased demand for renewable energy solutions.^1,2^ Among them, bioenergy is a sustainable alternative to conventional energy sources. It is derived from biomass and enables the production of various products, including biohydrogen, biogas, bio-oil, biochar, bioethanol, biodiesel, and syngas.^3–5^ Bioethanol is the most widely used transportation biofuel due to its high-octane number. It can be blended with gasoline or used as a stand-alone vehicle fuel.^6^ The United States is the largest ethanol producer, contributing 52% of global output, followed by Brazil with 28%. In the 2024 harvest, global ethanol production reached a total of 118.17 billion liters.^7^

Conventional crops such as sugar cane, corn, cassava, sorghum, and other grains can be used as feedstock to produce bioethanol through microbial fermentation.^8^ Grain sorghum is a starch-rich cereal similar to corn and well-suited for bioethanol production. It offers agronomic advantages, including adaptability to various soils and climates, efficient water use, and drought tolerance.^9^ Currently, 11.1 million tons of sorghum are produced in the U.S., with around one-third of the total sorghum being used for ethanol production.^10^

Given its potential for biofuel applications, previous studies on sorghum-based ethanol production have primarily focused on feedstock characteristics and yeast strain selection to improve ethanol concentration and yield.^11–13^ For instance, Weiss et al.^14^ evaluated different sorghum varieties and yeast strains, reporting that waxy sorghum combined with Ethanol Red yeast resulted in the highest ethanol concentrations and yields. While these approaches improve fermentation performance, they do not address ethanol accumulation during fermentation, which remains a key process limitation at high solid loadings.

The dry-grind process is the most common method for producing sorghum ethanol, and plants typically operate with a solid slurry concentration of 25–32% w w^–1^. The process includes size reduction, liquefaction, saccharification, fermentation, distillation, and coproduct recovery, yielding dried distiller’s grain with solubles (DDGS) and wet distiller’s grain with solubles (WDGS).^15^ The fermentation process can be performed either sequentially to saccharification, known as separated hydrolysis and fermentation (SHF), or through simultaneous saccharification and fermentation (SSF), in which the hydrolysis is carried out progressively throughout the process. The released glucose is directly metabolized, avoiding osmotic stress and growth inhibition.^16–18^ At the end of the fermentation process, the ethanol content reaches around 12–15% v v^–1^. Ethanol accumulation in the fermentative broth is a critical factor that can affect fermentation due to its inhibitory effect on yeast metabolism. One approach to overcome the inhibitory effects is extractive fermentation, which includes several techniques (such as gas stripping, liquid–liquid extraction, pervaporation, and vacuum) that have been investigated for ethanol recovery from the broth during its production.^15,19–22^ These techniques are typically evaluated based on parameters such as ethanol removal rate and product selectivity. However, their application in fermentation systems remains limited owing to challenges such as solvent toxicity, membrane fouling, and mass transfer limitations, particularly under high solid loadings.

Vacuum-assisted gas stripping has emerged as a promising technique for ethanol removal from fermentation broth because it enhances the mass transfer driving force by reducing system pressure while preserving operational simplicity and selectivity. Almeida et al.^23^ successfully applied vacuum-stripping for ethanol removal in preliminary tests using hydroalcoholic solutions. The authors achieved a gas stream six times more concentrated in ethanol than the liquid phase. However, its application in real fermentation systems, particularly in extractive SHF and SSF processes, remains largely unexplored. In this context, the application of vacuum-assisted gas stripping is still limited, especially under industrially relevant conditions. Moreover, its integration with fermentation kinetics and the development of predictive models that account for in situ ethanol removal have not yet been sufficiently addressed.

This study investigates ethanol removal from the SHF and SSF processes using vacuum-assisted gas stripping. A fermentation model was developed, and kinetic parameters were determined through a model-based optimization algorithm. First, the kinetic parameters of saccharification and fermentation were determined individually. Then, SSF experiments were conducted to evaluate the predictive capacity of the proposed model. Finally, SHF and SSF experiments with ethanol removal using vacuum and gas stripping were carried out. The findings of this study highlight the potential of vacuum-assisted extractive fermentation as an effective process intensification strategy. Furthermore, the validated modeling approach provides a reliable framework for evaluating and comparing conventional and vacuum-assisted SHF and SSF configurations, incorporating the effects of in situ ethanol removal.

## 2 Materials and Methods

This study integrates experimental procedures and mathematical modeling to evaluate ethanol production under conventional and extractive conditions. First, sorghum slurry was prepared and enzymatically liquefied, followed by saccharification and fermentation under SHF and SSF configurations. Extractive fermentations using vacuum-assisted gas stripping were then conducted. Finally, the experimental data were used for model development, parameter estimation, and validation.

### 2.1 Sorghum, Enzymes and Microorganism

Grain sorghum was obtained from a commercial seed company (MBS Seed, Denton, TX). The enzymes α-amylase (Liquozyme) and glucoamylase (Spirizyme) were sourced from Novonesis (Bagsvaerd, Denmark). Dry yeast (*Saccharomyces cerevisiae*, Ethanol Red, Lesaffre, Milwaukee, WI) was used for ethanol fermentation.

### 2.2 Liquefaction

Grain sorghum was ground using a cyclone sample mill (Model 3010, Udy Corporation, Fort Collins, CO, USA) equipped with a 1.0 mm screen. A sorghum slurry with 25% w w^–1^ solid content (dry basis) was prepared in 250 mL Erlenmeyer flasks, each containing 100 mL working volume. Then, 20 μL of α-amylase enzyme (240 KNU/g starch, ∼1.26 g/mL) was added to each flask.

Liquefaction was carried out in a water-bath shaker (Model 939XL, Amerex Instruments, Concord, CA) at 170 rpm. The temperature was gradually increased from 70 to 90 °C over 30 min, then reduced to 85 °C and maintained for 60 min.^24^

### 2.3 Saccharification

#### 2.3.1 Experimental Procedure

Saccharification was performed at 32 °C and pH 4.2 for 12 h in Erlenmeyer flasks containing 100 mL of liquefied sorghum slurry. Each flask (run in duplicate) received 100 μL of glucoamylase enzyme (750 AGU/g, ∼1.15 g/mL). Samples were collected every 2 h to measure glucose concentration, and glucoamylase was deactivated by immersing samples in boiling water for 10 min. The activity of glucoamylase was defined as the amount of enzyme required to release 1 μmol of glucose per min, calculated based on the initial reaction rate.

#### 2.3.2 Mathematical Modeling of Saccharification

The saccharification model was developed based on mass balances for liquefied starch (St) and glucose (G). The corresponding governing equations for the system are given by eqs 1 and 2.

1 $$\frac{dC_{\mathrm{St}}}{dt}=-r_{S}$$

2 $$\frac{dC_{G}}{dt}=Y_{G/\mathrm{St}}\cdot r_{S}$$

where *C*_St_ is the liquefied starch concentration (g L^–1^), *C*_G_ is the glucose concentration (g L^–1^), *r*_S_ is the saccharification rate, and *Y*_G/St_ is the stoichiometric yield coefficient (g_G_ g_St_^–1^), assumed to be 1.11 based on the stoichiometric conversion of starch into glucose.^25^

The saccharification kinetics catalyzed by glucoamylase was described using Michaelis–Menten kinetics with competitive glucose inhibition.

3 $$r_{S}=\frac{k_{1}\cdot C_{Enz}\cdot C_{St}}{K_{m}\left(1+\frac{C_{G}}{K_{i}^{G}}\right)+C_{St}}$$

where *k*_1_ is the saccharification rate constant (g U^1–^ h^–1^), *K*_m_ is the Michaelis–Menten constant (g L^–1^), *K*_i_^G^ is the inhibition constant of glucoamylase by glucose (g L^–1^), and *C*_Enz_ is the enzyme concentration (U L^–1^).

Initial conditions for the saccharification model were: *C*_St_(0) = 166.0 g L^–1^ and C_G_(0) = 9.81 g L^–1^.

### 2.4 SHF and SSF

In the SHF assays, the pH of the liquefied sorghum slurry was first adjusted to 5.5 with 1 M HCl. Then, 100 μL of glucoamylase enzyme was added, and saccharification was performed at 65 °C for 12 h using a water-bath shaker at 170 rpm. After saccharification, the pH was adjusted to 4.2 with 1 M HCl (36.5–38.0%, Thermo Fisher Scientific). Subsequently, 1 mL of activated yeast culture (∼2.8 × 10^8^ cells mL^–1^), 0.1 g of KH_2_PO_4_ (≥99%, Thermo Fisher Scientific), and 0.3 g of yeast extract (Thermo Fisher Scientific) were added to each flask. Ethanol fermentations were carried out (in duplicate) at 32 °C for 72 h in a shaker incubator set at 150 rpm. The yeast culture was prepared by dispersing 1.0 g of dry yeast in 19 mL of culture medium, followed by incubation in a shaking incubator at 38 °C for 30 min with agitation set at 150 rpm. The culture medium consisted of 20 g L^–1^ glucose (≥99%, Thermo Fisher Scientific), 5.0 g L^–1^ peptone (Thermo Fisher Scientific), 3.0 g L^–1^ yeast extract (Thermo Fisher Scientific), 1.0 g L^–1^ KH_2_PO_4_ (≥99%, Thermo Fisher Scientific), and 0.5 g L^–1^ MgSO_4_·7H_2_O (≥99%, Thermo Fisher Scientific).

For SSF assays, yeast and glucoamylase enzyme were added at the same time to the liquefied sorghum slurry in the same amounts used for SHF assays. SSF assays were carried out (in duplicate) at 32 °C and pH 4.2 for 72 h in a shaker incubator set at 150 rpm. Samples (2 mL) were collected at various time intervals to monitor the fermentation process.

### 2.5 Extractive Ethanol Fermentations

Extractive ethanol fermentations for both SHF and SSF methods were performed in a bubble column pneumatic bioreactor (2–L working volume, 9.7 cm internal diameter, 33.8 cm liquid height, and 56.2 cm total height). The bioreactor pressure (at vacuum conditions) was maintained at 41.3 kPa by a vacuum pump, and CO_2_ was injected at 1.0 vvm through a perforated cross-sparger at the base of the bioreactor. The fermentation temperature was kept at 32 °C using a thermostatic bath connected to a water jacket on the bioreactor. Figure 1 shows the experimental apparatus used in the extractive fermentations.

**Figure 1:**
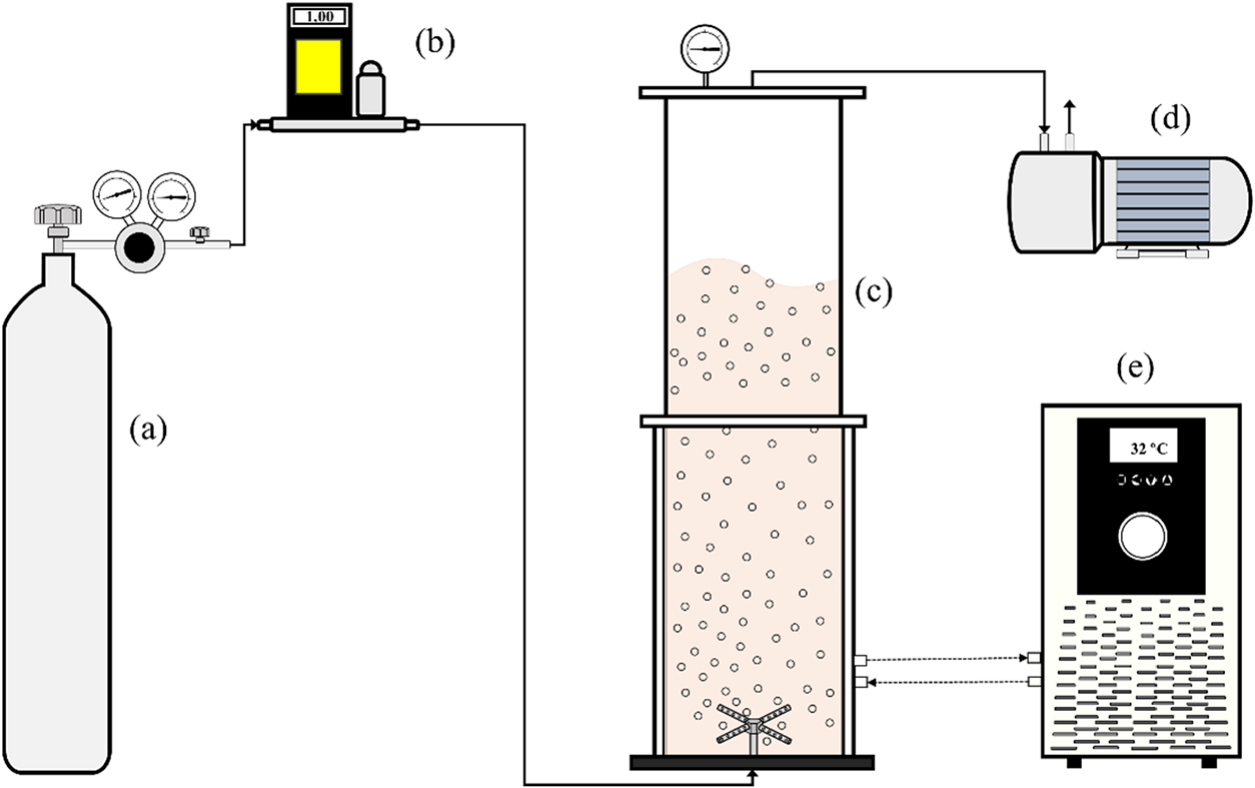
Schematic illustration of the experimental apparatus used in the extractive fermentations: (a) CO_2_ cylinder, (b) mass flow controller, (c) bioreactor, (d) vacuum pump, and (e) thermostatic bath.

### 2.6 Analytical Methods

The moisture content of sorghum was determined using the AOAC 930.15 standard method. Starch content was analyzed with the Megazyme Total Starch Assay Kit, following AACC method 79–13. Glucose and ethanol concentrations were measured by high-performance liquid chromatography (HPLC) using an Agilent system (1200 series, Santa Clara, CA) equipped with a refractive index detector operated at 45 °C, and an HPX-87H organic acid column (7.8 × 300 mm^2^) maintained at 60 °C. The mobile phase consisted of 5 mM H_2_SO_4_ at a flow rate of 0.60 mL min^–1^. Cell density was not directly measured due to the presence of suspended solids in the sorghum slurry, which hinders accurate biomass quantification using conventional methods. Therefore, fermentation performance was analyzed based on substrate consumption and product formation profiles.

### 2.7 Mathematical Modeling of Conventional and Extractive Ethanol Fermentation

Mathematical models were developed to describe conventional and extractive fermentation for SHF and SSF processes.

#### 2.7.1 SHF

The extractive SHF model was developed based on the mass balances for total cells (X) (eq 4), glucose (G) (eq 5), and ethanol (E) (eq 6), while also accounting for changes in broth volume (eq 7).

4 $$\frac{dC_{X}}{dt}=\mu \cdot C_{X}-\frac{C_{X}}{V}\cdot \frac{dV}{dt}$$

5 $$\frac{dC_{G}}{dt}=-\frac{1}{Y_{X/G}}\mu \cdot C_{X}-\frac{C_{G}}{V}\cdot \frac{dV}{dt}$$

6 $$\frac{dC_{E}}{dt}=\frac{Y_{E/G}}{Y_{X/G}}\cdot \mu \cdot C_{X}-k_{E}\cdot C_{E}-\frac{C_{E}}{V}\cdot \frac{dV}{dt}$$

7 $$\frac{dV}{dt}=-\frac{\left(k_{E}\cdot C_{E}+k_{W}\cdot \left(\rho_{W}-C_{E}\right)\right)\cdot V}{\rho_{W}}$$

where μ is the specific cell growth rate (h^–1^), *C*_X_ is the total cell concentration (g L^–1^), *C*_G_ is the glucose concentration (g L^–1^), *C*_E_ is the ethanol concentration (g L^–1^), *Y*_X/G_ represents the cell yield coefficient (g_X_ g_G_^–1^), *Y*_E/G_ denotes the ethanol yield coefficient (g_E_ g_G_^–1^), ρ_W_ is the specific mass of water (g L^–1^), and *k*_E_ and *k*_W_ are the removal rate constants for ethanol and water, respectively (h^–1^).

The specific cell growth rate (μ) was modeled using the hybrid Andrews-Levenspiel kinetic equation,^26,27^ which accounts for inhibition by substrate (glucose) and product (ethanol)

8 $$\mu =\mu_{\max}\cdot \frac{C_{G}}{K_{S}+C_{G}+\frac{C_{G}^{2}}{K_{\mathrm{IG}}}}\cdot {\left(1-\frac{C_{E}}{C_{\mathrm{Emax}}}\right)}^{n}$$

where μ_max_ is the maximum specific cell growth rate (h^–1^), *K*_S_ is the saturation constant (g L^–1^), *K*_IG_ is the glucose inhibition constant (g L^–1^), *C*_Emax_ is the ethanol concentration at which cell growth ceases (g L^–1^), and *n* is a dimensionless parameter related to the ethanol’s toxic potential.

The cell and ethanol yield coefficients, *Y*_X/G_ and *Y*_E/G_, were determined using the following equations

9 $$Y_{X/G}=\frac{C_{\mathrm{Xf}}-C_{X0}}{C_{G0}-C_{\mathrm{Gf}}}$$

10 $$Y_{E/G}=\frac{C_{\mathrm{Ef}}-C_{E0}}{C_{G0}-C_{\mathrm{Gf}}}$$

where the subscripts “0” and “f” denote the initial and final times of the culture, respectively.

The initial conditions for the SHF model were: *C*_X_(0) = 2.27 g L^–1^, *C*_G_(0) = 195.0 g L^–1^, *C*_E_(0) = 0 g L^–1^. For extractive conditions, the initial reactor volume was defined as V(0) = 2.0 L.

In the conventional SHF modeling, the constants *k*_E_ and *k*_W_ were set to zero (*k*_E_ = *k*_W_ = 0) because there was no removal of ethanol and water. For the extractive fermentations, *k*_E_ and *k*_W_ were calculated using equations proposed by Rodrigues et al.^28^

#### 2.7.2 SSF

The model for extractive SSF was developed based on mass balances for total cells (eq 11), liquefied starch (St) (eq 12), glucose (eq 13), and ethanol (eq 14), while accounting for changes in the broth volume (eq 15).

11 $$\frac{dC_{X}}{dt}=\mu \cdot C_{X}-\frac{C_{X}}{V}\cdot \frac{dV}{dt}$$

12 $$\frac{dC_{\mathrm{St}}}{dt}=-\frac{k_{1}\cdot C_{\mathrm{Enz}}\cdot C_{\mathrm{St}}}{K_{m}\left(1+\frac{C_{G}}{K_{i}^{G}}\right)+C_{\mathrm{St}}}-\frac{C_{\mathrm{St}}}{V}\cdot \frac{dV}{dt}$$

13 $$\frac{dC_{G}}{dt}=Y_{G/\mathrm{St}}\cdot \frac{k_{1}\cdot C_{\mathrm{Enz}}\cdot C_{\mathrm{St}}}{K_{m}\left(1+\frac{C_{G}}{K_{i}^{G}}\right)+C_{\mathrm{St}}}-\frac{1}{Y_{X/G}}\cdot \mu \cdot C_{X}-\frac{C_{G}}{V}\cdot \frac{dV}{dt}$$

14 $$\frac{dC_{E}}{dt}=\frac{Y_{E/G}}{Y_{X/G}}\cdot \mu \cdot C_{X}-k_{E}\cdot C_{E}-\frac{C_{E}}{V}\cdot \frac{dV}{dt}$$

15 $$\frac{dV}{dt}=-\frac{\left(k_{E}\cdot C_{E}+k_{W}\cdot \left(\rho_{W}-C_{E}\right)\right)\cdot V}{\rho_{W}}$$

In conventional SSF modeling, the constants *k*_E_ and *k*_W_ were set to zero (*k*_E_ = *k*_W_ = 0) because there was no removal of ethanol and water.

The initial conditions for the SSF model were: *C*_X_(0) = 2.27 g L^–1^, *C*_St_(0) = 166.0 g L^–1^, *C*_G_(0) = 9.73 g L^–1^, *C*_E_(0) = 0.0 g L^–1^. For extractive conditions, the initial reactor volume was defined as V(0) = 2.0 L.

### 2.8 Parameters Estimation and Model Validation

Modeling and simulation were performed using Scilab software (version 6.0.2). The system of differential equations describing saccharification, SHF, and SSF was solved numerically using the Runge–Kutta method with the initial conditions defined in Sections 2.3 and 2.7.

Parameter estimation was performed by minimizing the objective function E­(θ), defined in eq 16, to fit the model predictions to the experimental data. The fitting procedure was based on substrate and ethanol concentration data, as biomass measurements were not considered due to experimental limitations arising from the solid fraction present in the fermentation medium.

16 $$E\left(\theta \right)=\sum_{k=1}^{n}\left[\sum_{j=1}^{m}{\left(\frac{C_{j,k}-{Ĉ}_{j,k}}{C_{j,h}}\right)}^{2}\right]$$

where θ is the vector of parameters to be estimated, *n* is the number of experimental data points collected in each assay, m is the number of variables considered, and j denotes the concentration variables considered in the model. *C*_j,k_, is the experimental concentration for the *k* ^th^ sample, *Ĉ*_j,k_ is simulated concentration for the *k* ^th^ sample, and *C*_j,h_ is the highest experimental concentration values.

The kinetic parameters estimated in this study included *k*_1_, *K*_i_^G^, *K*_m_, μ_max_, *K*_S_, *K*_IG_, *C*_Emax_, and n. The remaining parameters and initial conditions (*C*_Enz_, *C*_St0_, *C*_G0_, *C*_E0_, *C*_X0_, Y_X/G_, *Y*_E/G_, *k*_E_, and *k*_W_) were defined based on experimental conditions. The biomass yield coefficient (*Y*_X/G_) was determined independently using clarified hydrolysate to ensure reliable quantification (data not shown). The bounds used for parameter estimation were defined based on prior knowledge and physical plausibility and are summarized in Table 1.

**Table 1:** Definition, Units, and Search Bounds of the Parameters Estimated Using a Genetic Algorithm

estimated parameter	description	unit	bounds
*k*_1_	saccharification rate constant	g U^1–^ h^–1^	[0.01, 0.99]
*K*_m_	Michaelis–Menten constant	g L^–1^	[5.0, 20.0]
*K*_*i*_^*G*^	glucose inhibition constant for saccharification	g L^–1^	[0.1, 10.0]
μ_max_	maximum specific growth rate	h^–1^	[0.05, 0.25]
*K*_S_	saturation constant	g L^–1^	[50.0, 100.0]
*K*_IG_	glucose inhibition constant	g L^–1^	[100.0, 200.0]
*C*_Emax_	ethanol concentration for complete growth inhibition	g L^–1^	[85.0, 120.0]
*n*	ethanol inhibition exponent	^–^	[0.1, 0.9]

### 2.9 Statistical and Sensitivity Analysis

The agreement between model predictions and experimental data was assessed using the coefficient of determination (*R* ^2^) and the root-mean-square error (RMSE) for each experimental condition, as defined in eqs 17 and 18. RMSE represents the average magnitude of the prediction error, while R^2^ indicates the proportion of variance in the experimental data explained by the model.

17 $$\mathrm{RMSE}=\sqrt{\frac{1}{n}\sum_{k=1}^{n}{\left({Ĉ}_{k}-C_{k}\right)}^{2}}$$

18 $$R^{2}=1-\frac{\sum_{k=1}^{n}{\left(C_{k}-{Ĉ}_{k}\right)}^{2}}{\sum_{k=1}^{n}{\left(C_{k}-\mathrm{C̅}\right)}^{2}}$$

where *Ĉ*_k_ and *C*_k_ are the estimated and experimental concentrations, respectively, and *C̅* is the mean of the experimental concentrations.

In addition, a local sensitivity analysis was performed to evaluate the influence of kinetic parameters on the model-predicted glucose (*C*_G_) and ethanol (*C*_E_) concentrations. A one-at-a-time (OAT) approach was adopted, in which each parameter was individually perturbed by ± 5% around its estimated value while the remaining parameters were kept constant. Sensitivity coefficients were computed using normalized central finite differences, yielding time-dependent sensitivity profiles that describe the evolution of parameter influence throughout the process. The normalized sensitivity coefficient was calculated as

19 $$S_{C_{j},P_{i}}=\frac{1}{n}\sum_{k=1}^{n}\left|\frac{{Ĉ}_{j}\left(t_{k},P_{i}\left(1+0.05\right)\right)-{Ĉ}_{j}\left(t_{k},P_{i}\left(1-0.05\right)\right)}{0.10\cdot {Ĉ}_{j}\left(t_{k},P_{i}\right)}\right|$$

where *Ĉ*_j_ (*t*_k_,*P*_i_) represents the model prediction for variable *C*_j_ at time *t*_k_ using the reference parameter set, and *Ĉ*_j_(*t*_k_,*P*_i_ (1 ± 0.05))­corresponds to the predictions obtained after a ± 5% perturbation in parameter *P*_i_.

## 3 Results and Discussion

### 3.1 Estimation of Kinetic Parameters for the Saccharification

The Michaelis–Menten equation is the fundamental model used to understand enzyme kinetics in the absence of inhibition mechanisms. When inhibitors are present, additional terms must be incorporated into the Michaelis–Menten model to accurately represent the process. Numerous studies have examined enzyme inhibition caused by high product concentrations during saccharification.^29–31^ Among these, glucose competitive inhibition has been identified as the predominant mechanism,^32–34^ although other mechanisms, such as noncompetitive and uncompetitive inhibition, have also been explored.

In this study, the kinetics of saccharification catalyzed by the glucoamylase enzyme were modeled using an unstructured mathematical model (eqs 1 and 2) based on Michaelis–Menten kinetics and incorporating competitive glucose inhibition. The sorghum biomass used in the saccharification assays contained 70.27% starch (w/w, dry basis). Figure 2 presents the time-course profile of glucose generated from the hydrolysis of liquefied starch. The coefficient of determination (*R* ^2^ = 0.97) indicates strong agreement between the experimental and simulated glucose profiles. Furthermore, the RMSE (9.60 g L^–1^) confirms that deviations between predicted and experimental values remain relatively low. A detailed comparison between experimental and simulated glucose concentrations is provided in Table S1 (Supporting Information).

**Figure 2:**
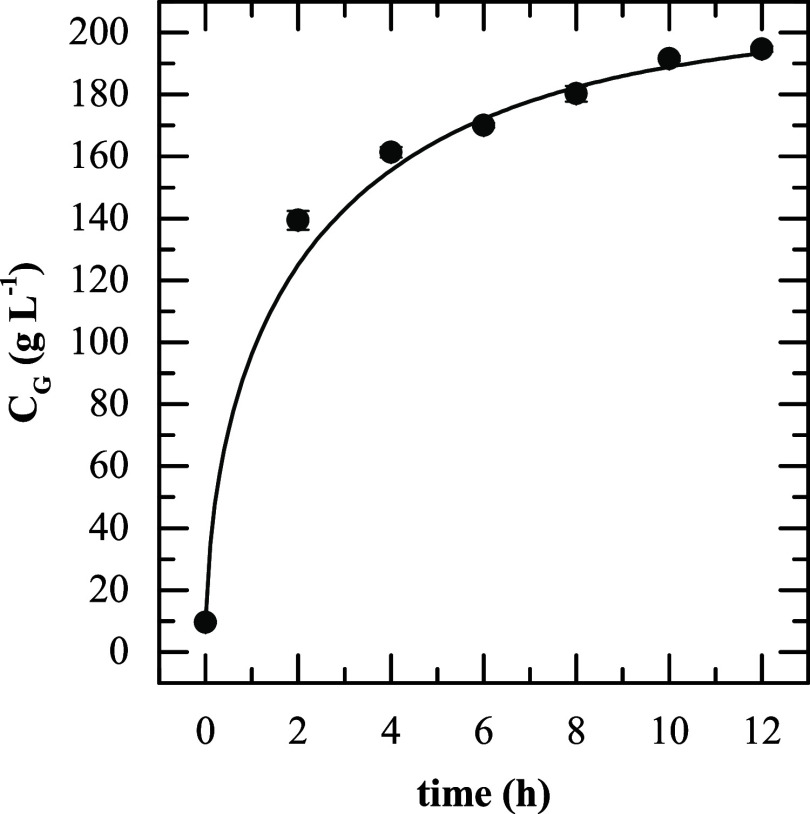
Simulated (lines) and experimental (points) glucose concentration profiles in the saccharification assay (100 mL working volume). Error bars correspond to the standard deviation.

Table 2 shows the kinetic parameters obtained for the saccharification model. The values of *K*_m_, *k*_1_, and *K*_i_^G^ were in good agreement with data previously reported in the literature.^32,34^ The low value of glucose inhibition constant (*K*_i_^G^ = 0.31 g L^–1^) indicates that the glucoamylase enzymatic reaction was strongly inhibited by glucose. Similar findings were reported by Polakovič and Bryjak,^34^ who studied the hydrolysis kinetics of soluble potato starch catalyzed by glucoamylase.

**Table 2:** Values of Kinetic Parameters for Saccharification, Fermentation, Yield Coefficients and Vacuum-Stripping Parameters^a^

estimated parameter	value	unit
*k*_1_	0.85 ± 0.24	g U^1–^ h^–1^
*K*_m_	19.01 ± 5.67	g L^–1^
*K*_*i*_^*G*^	0.31 ± 0.09	g L^–1^
μ_max_	0.16 ± 0.01	h^–1^
*K*_S_	73.58 ± 16.03	g L^–1^
*K*_IG_	167.11 ± 24.21	g L^–1^
*C*_Emax_	94.34 ± 16.03	g L^–1^
*n*	0.81 ± 0.16	^–^

fixed parameter	value	unit
*C*_Enz_	2552.24	U L^–1^
*Y*_X/G_	0.027	g_X_ g_G_^–1^
*Y*_E/G_	0.404	g_E_ g_G_^–1^
*k*_E_	0.0672	h^–1^
*k*_W_	0.0088	h^–1^

a Estimated using a 95% confidence level (Student’s *t* test).

### 3.2 Conventional Fermentations

#### 3.2.1 Modeling of Separate Hydrolysis and Fermentation (SHF)

Figure 3 presents the time-course profile of glucose (*C*_G_) and ethanol (*C*_E_) concentrations during the SHF process. As can be seen, the initial glucose concentration was 195.60 ± 0.46 g L^–1^ and was completely depleted within 48 h. Ethanol production reached a final concentration of 78.90 ± 1.85 g L^–1^ and a productivity of 1.64 ± 0.03 g L^–1^ h^–1^ by the end of the fermentation process. The ethanol yield coefficient (*Y*_E/G_) was 0.404 ± 0.020 g_E_ g_G_^–1^ based on total available sugars, corresponding to 79.10% of the theoretical yield. These results are consistent with findings from Weiss et al.,^14^ who reported ethanol concentration values ranging from 79.81 to 89.47 g L^–1^ for similar ethanol fermentations from sorghum.

**Figure 3:**
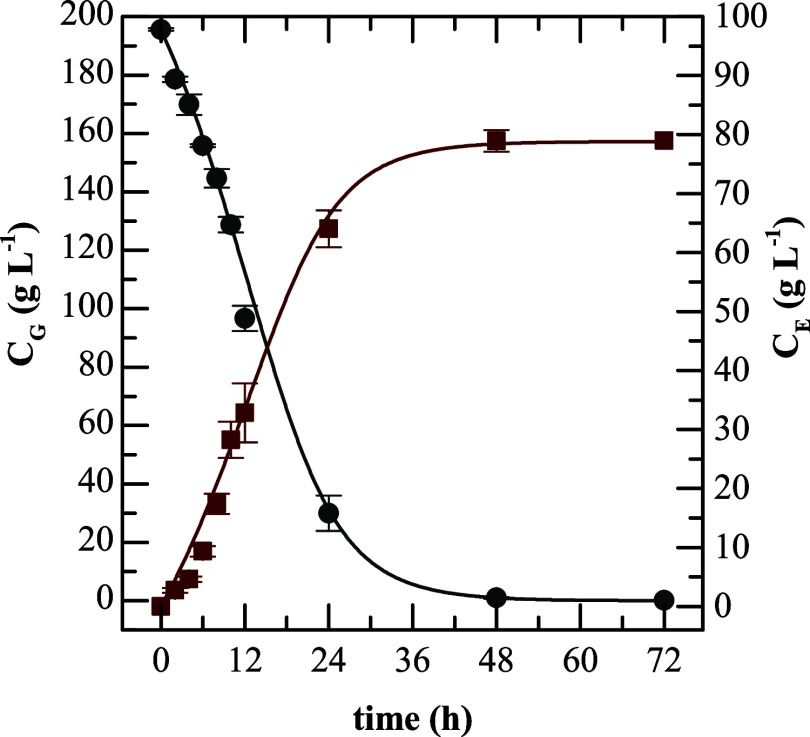
Simulated (lines) and experimental (points) concentration profiles in SHF experiments for glucose (circles) and ethanol (squares) (100 mL working volume). Error bars correspond to the standard deviation.

The cell yield coefficient (*Y*_X/G_) and ethanol yield coefficient (*Y*_E/G_) were calculated from experimental data for cell (*C*_X_), glucose (*C*_G_), and ethanol (*C*_E_) concentrations using eqs 8 and 9. The kinetic parameters of the Andrews-Levenspiel model (μ_max_, *K*_S_, *K*_IG_, *C*_Emax_, and n) were estimated using a genetic algorithm in conjunction with the Runge–Kutta method for numerically solving the set of differential equations (eqs 3–6), assuming *k*_E_ = *k*_W_ = 0. The criteria for obtaining kinetic parameters involved minimizing the sum of squared residuals (eq 15). The estimated kinetic parameter values that provided the best fit between the calculated and experimental data are shown in Table 2. The kinetic parameters are in close agreement with the range of values reported in the literature for processes conducted under experimental conditions similar to those used in this study.^35,36^

The comparison between simulated and experimental data demonstrated that the model provided an excellent fit, with R^2^ values of 0.99 for *C*_G_ and *C*_E_. Table 3 summarizes the statistical indicators obtained for all modeled systems, confirming the predictive capability of the model. A detailed comparison between experimental and simulated data for the SHF process is provided in Table S2 (Supporting Information).

**Table 3:** Statistical Indicators for Model Validation

process	variable	*R* ^2^	RMSE (g L^–1^)
saccharification	*C*_G_	0.97	9.61
SHF	*C*_G_	0.99	5.68
	*C*_E_	0.99	2.74
SSF	*C*_G_	0.99	6.23
	*C*_E_	0.98	3.66
ESHF	*C*_G_	0.99	1.83
	*C*_E_	0.98	2.33
ESSF	*C*_G_	0.99	2.31
	*C*_E_	0.99	1.89

Figure 4 illustrates the temporal profiles of the normalized sensitivity coefficients for ethanol concentration (*C*_E_), revealing a strong time-dependent parameter influence. At early times, μ_max_ dominates, whereas *C*_Emax_ becomes more relevant at intermediate stages, reflecting the increasing impact of product inhibition. In contrast, the parameters *K*_s_ and *n* exhibit negative sensitivities, and all parameters become negligible at later times as the system approaches substrate depletion.

**Figure 4:**
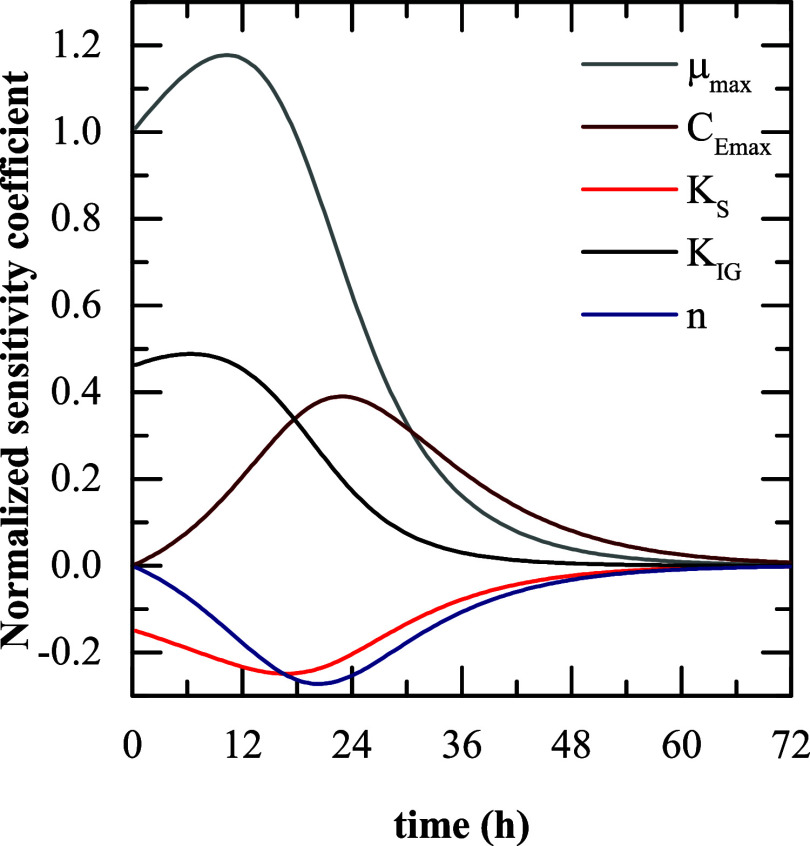
Temporal profiles of normalized sensitivity coefficients for *C*_E_.

#### 3.2.2 Modeling of Simultaneous Saccharification and Fermentation (SSF)

The development of mathematical models is a useful tool to simulate different strategies for design of efficient SSF process configurations.^37,38^ The kinetic parameters of the SSF model were determined through saccharification and SHF experiments, each of which individually investigates the kinetics of a specific step in the SSF process. Therefore, the kinetic parameters and yield parameters from saccharification (*K*_m_, *k*_1_, *K*_IG_) and fermentation (μ_max_, *K*_S_, *K*_IS_, *C*_Emax_, *n*, *Y*_X/G_, and *Y*_E/G_) processes were incorporated into the SSF model, along with the mass balance equations (eqs 10–14).

Figure 5 shows the experimental and simulated concentration profiles of glucose (*C*_G_) and ethanol (*C*_E_) for simultaneous saccharification and fermentation (SSF). The model demonstrates a good fit with the experimental data, with *R* ^2^ value of 0.99 for *C*_G_ and 0.99 for *C*_E_, and RMSE values of 6.23 g L^–1^ for *C*_G_ and 3.66 g L^–1^ for *C*_E_. Table S3 (Supporting Information) provides a comprehensive comparison between experimental and simulated data for the SSF process. The results could potentially be improved by directly fitting the parameters to the SSF experimental data. However, this study aimed to model the SSF process using kinetic parameters that were previously determined from batch experiments.

**Figure 5:**
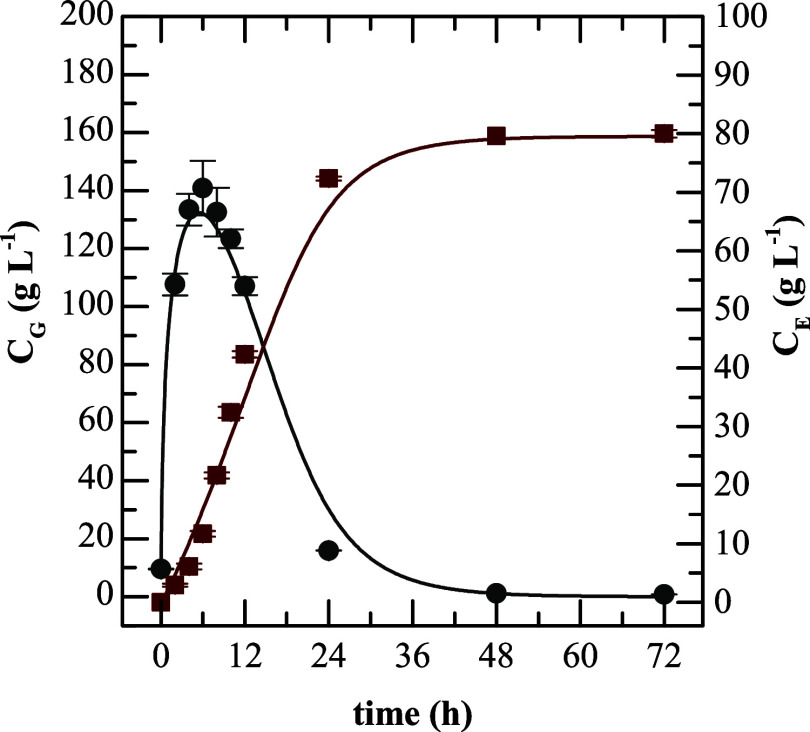
Simulated (lines) and experimental (points) concentration profiles in SSF experiments for glucose (circles) and ethanol (squares) (100 mL working volume). Error bars correspond to the standard deviation.

As shown in Figure 5, glucose concentration (*C*_G_) was completely depleted within 48 h, the ethanol concentration (*C*_E_) reached 79.54 ± 0.64 g L^–1^, and volumetric ethanol productivity (Pr_E_) was 1.66 ± 0.01 g L^–1^ h^–1^.

### 3.3 Modeling of Extractive SHF and SSF Process

The SHF and SSF models with ethanol removal by CO_2_ stripping were applied to evaluate the effect of ethanol removal on the dynamics of the extractive separate hydrolysis and fermentation (ESHF) and extractive simultaneous saccharification and fermentation (ESSF), using the values of removal rate constants for ethanol (*k*_E_) and water (*k*_W_) and the kinetic parameters for saccharification and fermentation. It is important to note that the *k*_E_ and *k*_W_ parameters used in the simulations were determined through vacuum-assisted stripping experiments conducted using an ethanol solution and provide a good approximation of ethanol removal in fermentation broth.

In the subsequent step, ESHF and ESSF experiments were performed to assess whether the model accurately represented the dynamics of extractive fermentation. Figure 6 (a,b) compares the experimental (symbols) and simulated (lines) concentration profiles of *C*_G_ and *C*_E_ obtained from the extractive experiments conducted in a 2–L bubble bioreactor. The proposed model accurately reproduced the behavior of both fermentations using the kinetic parameters obtained from experiments performed at a smaller scale (100 mL) without ethanol removal. The coefficient of determination (*R* ^2^) obtained for the ESHF was 0.99 for *C*_G_ and 0.98 for *C*_E_, whereas for the ESSF process it was 0.99 for both variables, with low RMSE values (Table 3). A detailed numerical comparison between experimental and simulated data for the extractive processes is provided in Tables S4 and S5 (Supporting Information). These results support the robustness of the proposed modeling framework, indicating that the same set of kinetic parameters was able to describe both conventional and extractive fermentations with good accuracy. Furthermore, the absence of systematic deviations between predicted and experimental profiles suggests that ethanol stripping did not significantly alter intrinsic yeast behavior within the evaluated operating range.

**Figure 6:**
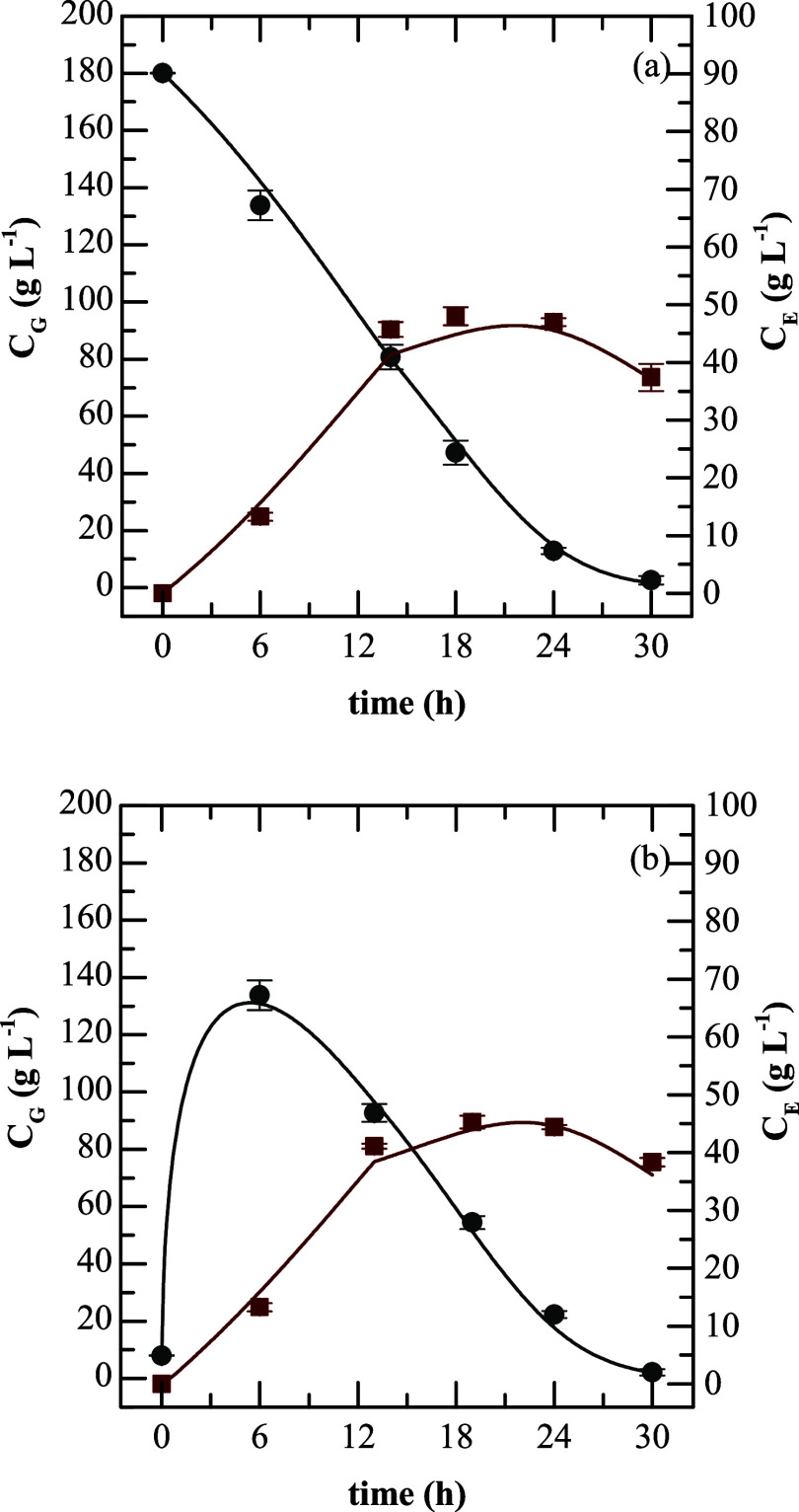
Simulated (lines) and experimental (points) concentration profiles for glucose (circles) and ethanol (squares) with ethanol removal using vacuum-stripping (2 L working volume): (a) ESHF and (b) ESSF. Error bars correspond to the standard deviation.

Ethanol removal using vacuum stripping was initiated once the ethanol concentrations (*C*_E_) reached approximately 40.0 g L^–1^, preventing the ethanol inhibitory effect from impacting yeast cell growth. According to Aiba et al.,^39^ yeast inhibition by ethanol becomes more pronounced once the ethanol concentration rises above 40.0 g L^–1^. Lemos et al.^21^ reported similar ethanol concentration in the fermentation broth (∼40.0 g L^–1^) for initiating ethanol removal in liquid–liquid extractive fed-batch ethanol fermentation. Additionally, Sonego et al.^40^ also reported a similar value (*C*_E_ = 35.0 g L^–1^) for beginning ethanol removal in fed-batch fermentations using CO_2_ stripping, with the ethanol concentration determined through an optimization algorithm.

In the extractive fermentations (ESHF and ESSF), glucose consumption occurred earlier than in the conventional fermentations (SHF and SSF). Glucose exhaustion was observed within 30 h for ESHF and ESSF, whereas in the conventional fermentations (SHF and SSF), depletion occurred at 48 h. This behavior can be attributed to the reduced inhibition of yeast activity caused by ethanol accumulation, as ethanol was removed throughout the fermentation process via vacuum stripping. In this technique, the gas phase is sparged in fermentation broth, and ethanol is transferred from the liquid to the gas phase until carbon dioxide bubbles become ethanol-saturated.^19^ Under vacuum conditions, the gas phase is richer in ethanol and water, as the vacuum lowers the saturation temperature, allowing for greater removal of ethanol and water.^41^

As shown in Table 4, the maximum ethanol concentrations in the liquid phase for ESHF and ESSF remained below 50.0 g L^–1^, highlighting the effective removal of ethanol. The ethanol productivities for ESHF and ESSF were up to 2.4 g L^–1^ h^–1^ as obtained by the ratio of the total final *C*_E_ and the time for substrate depletion. Therefore, the extractive fermentations had ethanol productivity (*P*_E_) values of around 60% higher than the conventional methods (SHF and SSF). Previous studies on ethanol extractive fermentation from corn starch have demonstrated high ethanol productivity and effective conversion of concentrated glucose feedstocks, using gas stripping^42^ or vacuum^15^ for ethanol removal. Although variability in process conditions limits direct comparisons, these findings support the results obtained in the present study.

**Table 4:** Performance Comparison of Conventional and Extractive Fermentations

variable	unit	fermentation			
		SHF	SSF	ESHF	ESSF
total *C*_G_ consumed	g L^–1^	195.29	195.00^a^	180.01	195.00^a^
maximum *C*_E_ in the broth	g L^–1^	78.90	79.08	47.30	45.29
total *C*_E_ at the end of fermentation	g L^–1^	78.90	79.08	73.12^b^	78.78^b^
ethanol productivity (*P*_E_)	g L^–1^ h^–1^	1.64	1.65	2.43	2.62

a Calculated considering starch content of 70.27%.

b Calculated considering *Y*_E/G_ (g_E_ g_G_^–1^) = 0.404.

Within each process configuration, ethanol productivities showed similar values, with comparable results observed between SHF and SSF, and likewise between ESHF and ESSF (Table 4). From a process perspective, the SSF configuration offers an advantage by reducing the number of required vessels, which can lower the initial investment costs. Moreover, heating costs are minimized since the maintenance of slurry temperatures during an independent saccharification stage is not required. Additionally, SSF prevents substrate inhibition caused by high glucose concentrations, as the yeast immediately converts the glucose generated during saccharification into ethanol.^18^ It is important to highlight that ethanol concentration in the broth is lower in extractive fermentations due to the ethanol removal by vacuum stripping. Nonetheless, the vaporized ethanol fraction can be recovered through techniques such as condensation,^43,44^ absorption,^45,46^ and adsorption.^47^ Although this study does not address ethanol recovery techniques, they are critical for improving ethanol production efficiency.

Overall, the experimental and modeling results indicate that integrating vacuum-assisted gas stripping into SHF and SSF modifies fermentation behavior by limiting ethanol accumulation and reducing inhibitory effects on yeast metabolism. In addition, the removal of ethanol and water changes the volume and composition of liquid streams entering subsequent separation steps, potentially reducing the load associated with downstream drying operations. Although an energy analysis was beyond the scope of this study, the findings support vacuum-assisted extractive fermentation as a relevant process intensification strategy for starch-based bioethanol production. Similarly, further investigation is needed to assess model performance at larger scales, where gas–liquid hydrodynamics, as well as concentration and temperature gradients, may become more significant. Under such conditions, spatial variations in substrate, product, and temperature can lead to nonuniform microbial exposure to different environmental conditions, affecting growth kinetics and fermentation performance in ways that may not be fully captured by the kinetic model developed under well-mixed assumptions. Therefore, incorporating scale-dependent effects may be necessary to improve the model’s predictive capability at industrial scales.

## 4 Conclusions

This study evaluated vacuum-assisted gas stripping for ethanol removal in SHF and SSF processes, combining experimental investigation with mathematical modeling. The results demonstrated that in situ ethanol removal effectively reduced product inhibition, leading to significant improvements in fermentation performance, with volumetric ethanol productivity increases of up to 60% compared to conventional systems.

The proposed mass-balance-based model successfully described the behavior of both conventional and extractive fermentations at bench scale, showing strong agreement with experimental data (average R^2^ = 0.99) and confirming its reliability as a tool for analyzing process configurations and optimization strategies.

Overall, these findings highlight the strong potential of vacuum-assisted extractive fermentation as a process intensification strategy for starch-based bioethanol production. Further studies addressing scale-up, process integration, and economic feasibility are required to assess its industrial applicability.

## Supplementary Material



## Data Availability

All data supporting the findings of this study are available within the article and its Supporting Information.

## Notes

Comparison between experimental and simulated glucose concentration during the saccharification (Table S1); experimental and simulated glucose and ethanol concentrations for the SHF process (Table S2), SSF process (Table S3); ESHF process (Table S4); and ESSF process (Table S5) (PDF)
