# Supplementary material for: Vacuum-Assisted Gas Stripping Extractive Fermentation for Process Intensification of Sorghum-Based Bioethanol Production: An Experimental and Modeling Study
Source: ACS Omega. 2026 Jun 29;11(27):40062–71. doi: 10.1021/acsomega.6c01889 (PMC13382827; doi:10.1021/acsomega.6c01889)
Supplement: Supplementary file 1 [file ao6c01889_si_001.pdf]

Supporting Information

Vacuum-assisted gas stripping extractive fermentation for process intensification of sorghum-based bioethanol production: An experimental and modeling study

Letícia P. Almeida[a], Mateus N. Esperança[b], Antonio J. G. Cruz[a], Mark R. Wilkins[c], Alberto C. Badino[a*]

[a] Graduate Program of Chemical Engineering, Federal University of São Carlos, São Carlos, SP, 13565-905, Brazil.

[b] Federal Institute of Education, Science, and Technology of São Paulo, Campus Capivari, Capivari, SP, 13365-010, Brazil.

[c] Carl and Melinda Helwig Department of Biological and Agricultural Engineering, Kansas State University, Manhattan, KS, 66506, USA.

*Corresponding author: badinojr@ufscar.br

Table S1. Comparison between experimental and simulated glucose concentration (C_G) during the saccharification.

Time (h)	$C_{G,Exp}$	$C_{G,Sim}$
0	9.73 ± 0.33	9.73
2	149.37 ± 3.11	124.99
4	161.30 ± 1.68	155.56
6	170.19 ± 0.78	172.15
8	180.23 ± 2.57	182.32
10	191.63 ± 0.82	188.92
12	194.70 ± 0.94	193.33

Table S2. Experimental and simulated glucose (C_G) and ethanol (C_E) concentrations for the SHF process**.**

**Time (h)**	$\mathbf{C_{G,Exp}}$	$\mathbf{C_{G,Sim}}$	$\mathbf{C_{E,Exp}}$	$\mathbf{C_{E,Sim}}$
0	195.60 ± 0.46	195.60	0.00 ± 0.00	0.00
2	178.54 ± 0.93	184.40	2.71 ± 0.40	4.52
4	169.90 ± 3.58	172.03	4.61 ± 0.44	9.50
6	155.86 ± 0.57	158.52	9.34 ± 0.91	14.94
8	144.67 ± 3.16	143.95	17.39 ± 1.72	20.81
10	128.74 ± 2.66	128.50	28.24 ± 3.06	27.04
12	96.70 ± 5.02	112.45	32.82 ± 4.33	33.51
24	29.95 ± 6.10	31.18	64.02 ± 3.14	66.26
48	0.97 ± 0.32	1.02	77.98 ± 1.85	78.42
72	0.11 ± 0.02	0.03	79.02 ± 0.15	78.81

Table S3. Experimental and simulated glucose (C_G) and ethanol (C_E) concentrations for the SSF process.

Time (h)	$C_{G,Exp}$	$C_{G,Sim}$	$C_{E,Exp}$	$C_{E,Sim}$
0	9.73 ± 0.11	9.73	0.00 ± 0.00	0.00
2	107.65 ± 3.78	112.13	2.92 ± 0.24	4.68
4	133.41 ± 5.41	129.66	6.14 ± 0.49	10.08
6	140.83 ± 9.41	132.05	11.67 ± 0.48	15.84
8	132.60 ± 8.39	126.89	21.67 ± 0.51	21.96
10	123.40 ± 3.21	117.23	32.43 ± 0.97	28.35
12	107.10 ± 3.06	104.80	42.31 ± 0.56	34.91
24	15.92 ± 0.10	30.07	72.34 ± 0.35	67.21
48	1.14 ± 0.30	1.10	79.58 ± 0.65	79.08
72	0.73 ± 0.49	0.04	79.97 ± 0.24	79.52

Table S4. Experimental and simulated glucose (C_G) and ethanol (C_E) concentrations for the ESHF process.

Time (h)	$C_{G,Exp}$	$C_{G,Sim}$	$C_{E,Exp}$	$C_{E,Sim}$
0	180.10 ± 0.10	180.10	0.00 ± 0.00	0.00
6	133.83 ± 5.21	142.08	13.27 ± 0.49	15.71
14	80.70 ± 4.29	80.12	45.70 ± 0.51	41.36
18	47.30 ± 4.24	51.43	48.00 ± 0.97	44.83
24	12.90 ± 1.12	14.82	47.00 ± 0.36	45.54
30	2.60 ± 1.50	1.66	37.40 ± 0.65	37.13

Table S5. Experimental and simulated glucose (C_G) and ethanol (C_E) concentrations for the ESSF process.

Time (h)	$C_{G,Exp}$	$C_{G,Sim}$	$C_{E,Exp}$	$C_{E,Sim}$
0	8.00 ± 0.13	8.00	0.00 ± 0.10	0.00
6	133.83 ± 5.21	130.86	13.27 ± 0.69	15.95
14	92.70 ± 3.14	96.51	41.11 ± 0.45	38.42
18	54.45 ± 2.28	51.30	45.29 ± 1.11	43.99
24	22.34 ± 1.23	17.72	44.42 ± 0.36	44.61
30	2.10 ± 1.10	2.06	38.34 ± 0.75	36.17
